# Branched Chain Fatty Acids Reduce the Incidence of Necrotizing Enterocolitis and Alter Gastrointestinal Microbial Ecology in a Neonatal Rat Model

**DOI:** 10.1371/journal.pone.0029032

**Published:** 2011-12-14

**Authors:** Rinat R. Ran-Ressler, Ludmila Khailova, Kelly M. Arganbright, Camille K. Adkins-Rieck, Zeina E. Jouni, Omry Koren, Ruth E. Ley, J. Thomas Brenna, Bohuslav Dvorak

**Affiliations:** 1 Division of Nutritional Sciences, Savage Hall, Cornell University, Ithaca, New York, United States of America; 2 Department of Pediatrics, University of Arizona, Tucson, Arizona, United States of America; 3 Mead Johnson Nutrition, Evansville, Indiana, United States of America; 4 Department of Microbiology, Cornell University, Ithaca, New York, United States of America; Hôpital Robert Debré, France

## Abstract

**Introduction:**

Branched chain fatty acids (BCFA) are found in the normal term human newborn's gut, deposited as major components of vernix caseosa ingested during late fetal life. We tested the hypothesis that premature infants' lack of exposure to gastrointestinal (GI) BCFA is associated with their microbiota and risk for necrotizing enterocolitis (NEC) using a neonatal rat model.

**Methods:**

Pups were collected one day before scheduled birth. The pups were exposed to asphyxia and cold stress to induce NEC. Pups were assigned to one of three experimental treatments. DF (dam-fed) ; Control, hand-fed rat milk substitute ; BCFA, hand-fed rat milk substitute with 20%w/w BCFA. Total fat was equivalent (11%wt) for both the Control and BCFA groups. Cecal microbiota were characterized by 16S rRNA gene pyrosequencing, and intestinal injury, ileal cytokine and mucin gene expression, interleukin-10 (IL-10) peptide immunohistochemistry, and BCFA uptake in ileum phospholipids, serum and liver were assessed.

**Results:**

NEC incidence was reduced by over 50% in the BCFA group compared to the Control group as assessed in ileal tissue; microbiota differed among all groups. BCFA-fed pups harbored greater levels of BCFA-associated *Bacillus subtilis* and *Pseudomonas aeruginosa* compared to Controls. *Bacillus subtilis* levels were five-fold greater in healthy pups compared to pups with NEC. BCFA were selectively incorporated into ileal phospholipids, serum and liver tissue. IL-10 expression increased three-fold in the BCFA group versus Controls and no other inflammatory or mucosal mRNA markers changed.

**Conclusion:**

At constant dietary fat level, BCFA reduce NEC incidence and alter microbiota composition. BCFA are also incorporated into pup ileum where they are associated with enhanced IL-10 and may exert other specific effects.

## Introduction

**Figure 1:**
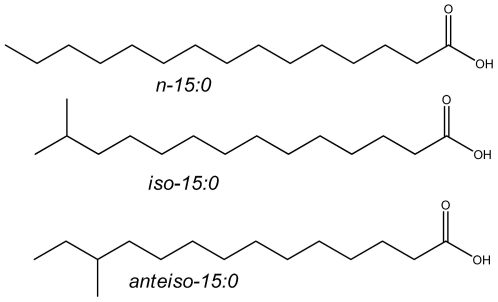
Structures of representative BCFA. *n- (normal)* hydrocarbon chains are straight with no branching. *iso-*BCFA have a bifurcated methyl branch. The systematic name for *iso*-15:0 is 13-methyl tetradecanoic acid. *Anteiso*-BCFA have a methyl branch on the antepenultimate carbon. *anteiso*-15:0 is 12-methyl tetradecanoic acid.

Branched chain fatty acids (BCFA) have one or more methyl branches on the carbon chain. Most methyl branching is at the ultimate (*iso*) or penultimate carbon (*anteiso*) (**Figure 1**). In humans, BCFA are synthesized mainly by the skin, and they are also found in colostrum and breast-milk at up to 1.5%w/w [1], [2]. Thus, BCFA are a natural component of breastfed infants' intake, at levels similar to long chain polyunsaturated fatty acids (FA) known to be highly bioactive. BCFA are prominent in ruminant meat and milk, constituting 2%w/w of cow milk fat in the US retail milk supply [3]. Finally, BCFA are a major component of the membranes of many bacteria [4], including *Lactobacilli* and *Bifidobacteria* [5], [6], which are present in the GI tract of the early infant [7], [8].

BCFA are found in vernix caseosa at the remarkably high level of 25–30%w/w [9]. Vernix is uniquely human, having been reported for no other mammals [10]; there is no information on the presence of BCFA in amniotic fluid (AF) in rats. Vernix production by human fetal skin begins about midway through normal gestation [11] and continues until term birth [12]. Late in gestation, vernix becomes suspended in AF, and is swallowed by the fetus in increasing amounts as term birth approaches [12], [13]. At term, AF contains about 154 mg/L lipids overall [14] and of these, BCFA are about 17 mg/L [15]. The fetus swallows 200–500 ml/d of AF near term [16], providing an estimated 6 mg BCFA per day exposure to the fetal gastrointestinal (GI) tract, and totaling 180 mg BCFA in the last month of gestation. BCFA are present in meconium of healthy term infants [15], implying that they persist through the length of the gut. Moreover, BCFA with fewer than 16 carbons were detected in vernix but not in meconium, while BCFA with at least 16 carbons were detected in both [15]. This selective shift in BCFA distribution indicates that the fetal alimentary canal metabolizes BCFA, suggesting that BCFA play a metabolic role in the developing gut.

Necrotizing enterocolitis (NEC) is a major cause of morbidity in premature infants with an estimated rate of death of 20–30% [17], [18]. Because NEC often progresses from early symptoms to extensive necrosis within hours, prophylactic measures are preferred [19], however none has emerged. The major risk factors include prematurity, enteral feeding, abnormal bacterial colonization, and intestinal hypoxia-ischemia [20], [21], though recent evidence suggests the latter is not a primary mechanism [22]. Despite showing some promise, nutritional interventions including minimal feeds [23] and probiotics [24] have been investigated experimentally with mixed results [25]. Human milk is associated with reduced NEC risk compared with formulas [26], [27], most of which do not contain BCFA. Other FA components of milk, in particular docosahexaenoic acid (DHA) and arachidonic acid (ARA), are protective against NEC [28], [29]. The incidence of NEC drops as gestational age approaches normal term [30], consistent with the increase in BCFA gut exposure from ingested vernix. If BCFA have a metabolically significant role in metabolism or gut colonization, a condition of BCFA deficiency would be expected. We speculate that development of NEC is related to the absence of BCFA, either from vernix, breast-milk, or both.

Abnormal gut colonization leading to excess gas production and necrosis is a hallmark of NEC [20], [21]. Term infants have more diverse GI bacteria than premature infants [31], who in turn have more diverse microbiota than premature infants with NEC, though no causative pathogen has emerged [32]. BCFA are prominent membrane components of many bacterial species [4], thus BCFA may protect against NEC by promoting the establishment of commensal BCFA-containing bacteria.

Numerous studies document the influence of probiotics and prebiotics on gut microbiota (e.g., [33], [34], [35]. Few studies have looked at the effect of specific nutrients on microbial ecology. Examples of these include digestion-resistant starch in adults [36], and iron fortification in anemic children aged 6–14 y old which supported growth of more pathogenic microbiota compared to a non-fortified diet [37]. Numerous investigations of high fat diets of indeterminate composition have demonstrated its effect on bacterial ecology of the gut [38], [39] but none report specifically on the effect of FA or classes of FA with total fat held constant on gut microbiota as in the present design, apart from a few studies of ruminants.

Here we test the hypothesis that BCFA reduce the incidence of NEC and systematically alter cecal microbiota of rats to favor BCFA-containing bacteria, using an established rat pup model in vivo. A BCFA mixture was substituted for 20%w/w of FA in normal rat pup feed to recapitulate and simulate ingestion of BCFA levels found in vernix. Microbiota profiling, ileal BCFA uptake and incorporation into phospholipids (PL), serum and liver, histological localization of interleukin-10 (IL-10), and gene expression (mRNA) for several inflammation-related cytokines were measured to explore the possible role of each in reducing NEC. We find that BCFA reduce the incidence of NEC by more then 50%, compared to the non-BCFA-fed controls. Furthermore, formula containing BCFA alter microbiota composition, supporting a greater abundance of the BCFA-containing bacteria *Bacillus subtilis* and *Pseudomonas aeruginosa*. BCFA were incorporated into ileal membrane PL and into pup serum and liver. The anti-inflammatory cytokine interleukin-10 (IL-10) mRNA triples in the ileum of BCFA-fed pups compared to controls.

## Materials and Methods

### Animal model and diets

Ethics Statement. The protocol was approved by the University of Arizona Animal Care and Use Committee (#A-324801-95081) and Cornell University.

Seventy-three neonatal Sprague-Dawley rats (Charles River Laboratory, Pontage, MI) were collected by caesarian section one day before scheduled birth. Gender is not routinely recorded because rats are born very immature and their gender is not obvious, the study is complete long before sex characteristics become dominant, and sex is not overtly related to NEC development in humans. Pups were assigned to one of three experimental groups: (a) dam-fed (DF, n = 15), (b) hand-fed with rat formula (Control, n = 35), and (c) hand-fed with rat formula prepared with 20%w/w of BCFA mixture (BCFA, n = 23). Body weights at the beginning of the study were 5.40±0.16, 5.44±0.08, and 5.49±0.16 g (means ± SEM) for the DF, Control and BCFA groups, respectively, and were not significantly different. Fewer rat pups are used in the DF group because none are lost due to treatment; fewer pups were used in the BCFA group than the control group because of the high cost of BCFA. Pups were drawn from eight litters in a balanced manner and randomly assigned to treatments; no litter effects were observed on outcomes (data/statistics not shown). The DF group is a control for general health of the newborn pups, and differs from the other two groups with respect to diet and maternal presence, which avoids exposure to the stress of artificial feeding.

The BCFA mixture had six FA in purified, free FA form (Larodan Fine Chemicals, Malmo, Sweden) in proportions similar to their mean proportions in vernix [15]: *iso*-14:0 (25%), *anteiso-*15:0 (20%), *iso-*16:0 (25%), *anteiso-*17:0 (8%), *iso-*18:0 (10%) and *iso-*20:0 (12%). These were chosen because they represent the central range of BCFA chain lengths found in vernix and are available commercially. The Control diet composition was described previously [40]. The BCFA diet was prepared as the Control diet, except that (a) the fat emulsion was prepared as described previously [41], but with BCFA added such that the complete diet contained 20%w/w BCFA, and (b) soy oil was used instead of almond oil to better match the FA composition of the artificial diets and to compensate for the soy oil exchanged for BCFA in the fat emulsion. Because they are expressed as a percent of total FA, oleic acid (18:1n-9) and linoleic acid (18:2n-6) levels differ between the diets due to the addition of BCFA (**Table 1**). Both oleic and linoleic acids are in excess compared to requirements and thus were reduced to accommodate BCFA and maintain constant percent energy as fat. Each pup in the artificial feeding groups received total diet of 850 µl/d.

**Table 1:** Fatty acid (FA) composition of Control and BCFA diets determined by gas-chromatography (GC) analysis.

FA	Control	BCFA
10:0	1.2	1.0
12:0	1.5	1.4
*iso-*14:0	0.1	4.9
14:0	4.8	4.8
14:1n-5	0.5	0.5
*anteiso-*15:0	0.2	3.9
15:0	0.5	0.5
*iso-*16:0	0.1	5.1
16:0	19.5	16.8
16:1	1.0	0.8
*anteiso-*17:0	0.2	1.6
17:0	0.3	0.3
*iso-*18:0	n.d.	1.9
18:0	8.5	8.0
18:1n-9	30.7	21.5
18:2n-6	27.1	20.8
*iso-*20:0	n.d.	2.5
18:3n-3	3.3	3.1
20:0	0.5	0.5
22:0	0.2	0.2

n.d., not detected.

### NEC induction

NEC was induced in all groups as described elsewhere [42], [43]. Briefly, pups were exposed to a hypoxia challenge in 100% nitrogen gas twice daily for 1 min, followed by hypothermia-exposure at 4°C for 10 min. After 96 h, all surviving pups were killed by decapitation.

**Figure 2:**
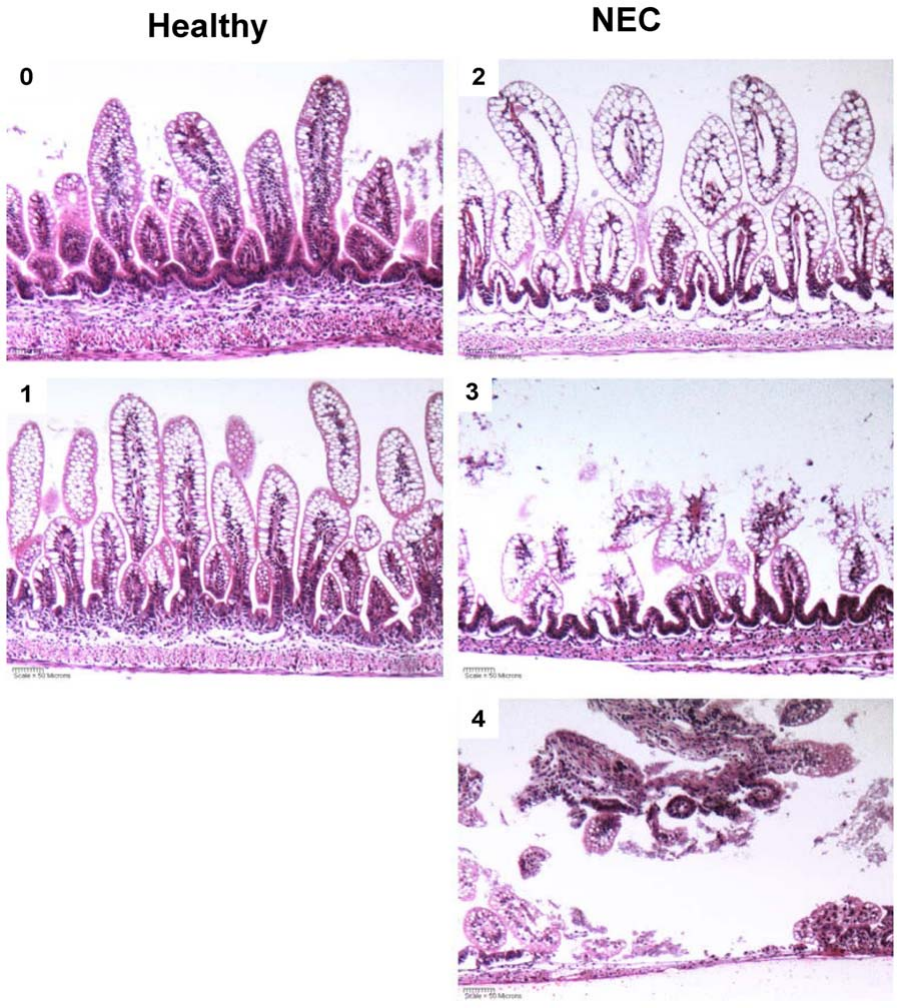
Representative histology of neonatal rat pup terminal ileum. Histological changes were graded as described in the methods section. Briefly: 0, normal ileum; 1–3, mild, moderate, or severe damage reflecting degrees of separation of submucosa and/or lamina propria, regional villous sloughing and initial necrosis; 4, necrosis. Pups were considered to have NEC if their histological score was ≥2. Magnification: 200×.

For microscopic evaluation, 2 cm of distal ileum were removed and processed as described previously [42]. A histopathologist masked to the treatments graded the histological changes using intestinal injury scores developed previously [44]: Grade 0, normal ileum; Grade 1, mild damage, slight submucosal and/or lamina propria separation; Grade 2, moderate to severe separation of the submucosa and/or lamina propria and/or edema in the submucosal and muscular layers, partial loss of villous sloughing; Grade 3, severe separation of the submucosa and/or lamina propria region, villous sloughing and initial villus necrosis; Grade 4, necrosis and loss of villi structure and/or transmural necrosis (**Figure 2**). Half grade increments (+0.5, 1.5, 2.5 and 3.5) were used to more accurately assess levels of ileal damage. Rat pups with histological scores of grade ≥2 were NEC positive; this value is used because normal DF pups, which never develop NEC, are always scored below this level.

### Rat RNA preparation and reverse transcription (RT) and real-time polymerase chain reaction (PCR)

Total RNA was isolated from ileal tissue (frozen in liquid N_2_) using the RNAeasy-Plus MiniKit (Qiagen, Santa Clarita, CA) as described in the manufacturer's protocol. Samples were incubated with RNAse-free DNAse (20 U/reaction) for 10 minutes at 37°C to eliminate DNA contamination. RNA was quantified by ultraviolet spectrophotometry, and purity was determined by the A260/A280 nm absorbance ratio (SPECTRAmax PLUS, Molecular Devices, Sunnyvale, CA). RNA purity and integrity was determined using a NanoDrop (Thermo Fisher Scientific, Wilmington, DE).

RT and real-time PCR assays were performed to quantify steady state mRNA levels of the following selected cytokines (IL-10, IL-18, interferon-*γ* (IFN-*γ*), tumor necrosis factor-*α* (TNF-*α*), transforming growth factor- *β* (TGF-*β*), Mucins (Muc 2, Muc 3, Muc 4) and trefoil factor 3 (TFF3). cDNA was synthesized from 0.5 µg of total RNA. Primers and probes were designed using Primer Express SoftwareTM (Applied Biosystems, Foster CA). Target probe was labeled with 6-carboxy-fluorescein (FAM) fluorescent reporter dye. The following sequences were used: Muc2 (GenBank BC036170): sense primer- 5′-actgggaatgtgactgctactg-3′; anti-sense primer- 5′-accctggtaactgtagtaaagtccat-3′; and probe- 5′-acaaagtgtgggtcccc-3′; TNF-*α* (GenBank X66539): sense primer- 5′-gtgatcggtcccaacaagga-3′; anti-sense primer- 5′-gggccatggaactgatgaga-3′; and probe- 5′-cccatttgggaacttc-3′. PreDeveloped TaqMan primers and probes were used for the detection of Muc 3, Muc 4, TFF3, IL-10, IL-18, IFN-*γ* and TGF-*β*. Reporter dye emission was detected by an automated sequence detector combined with ABI Prism 7700 Sequence Detection System® software (Applied Biosystems). Real time PCR quantification was then performed using TaqMan® 18S controls. mRNA levels are expressed as multiples of the values determined for the DF group and are presented as means ± SEM.

### DNA extraction and amplification

Thirty three cecal samples were used for bacterial analysis from the rat pups (DF, n = 10; Control, n = 10; BCFA, n = 13). The samples were processed as described previously [38]. Genomic DNA was isolated using the PowerSoil® DNA isolation kit (MoBio Laboratories Ltd, Carlsbad, CA). 16S rRNA genes were amplified by PCR from each of the 33 samples using a composite forward primer and a reverse primer containing a unique 12-base barcode [45] which was used to tag PCR products from respective samples [46]. We used the forward primer 5′- *GCCTTGCCA GCCCGCTCAGTC* **AGAGTTTGATCCTGGCTCAG** -3′ ′: the italicized sequence is 454 Life Sciences primer B, and the bold sequence is the broadly conserved bacterial primer 27F. The reverse primer was 5′-*GCCTCCCTCGCGCCATCAGNNNNNNNNNNNNCA* **TGCTGCCTCCCG TAGGAGT**-3′: the italicized sequence is 454 Life Sciences primer A, and the bold sequence is the broad-range bacterial primer 338R. *NNNNNNNNNNNN* designates the unique 12-base barcode used to tag each PCR product, with “CA” inserted as a linker between the barcode and rRNA gene primer. PCR reactions were carried out in triplicates and consisted of 2.5 U Easy-A high-fidelity enzyme and 1× buffer (Stratagene, La Jolla, CA), 200 µM of each primer, and the reaction conditions consisted of initial denaturation step at 95°C for 2 min followed by 30 cycles of denaturation at 95°C for 40 s, annealing at 57°C for 30 s, and extension step at 72°C for 1 min, and a final extension step at 72°C for 7 min. Replicate amplicons were pooled. A negative control sample was treated as described above, except that no template DNA was added to the PCR reaction. PCR amplicons were purified using a magnetic bead system (Mag-Bind® EZPure, Omega Bio-Tek, Norcross, GA) and the amplicons were quantified using the QuantiT PicoGreen dsDNA Assay Kit (Invitrogen, Carlsbad, CA). Aliquots of amplicons (at equal masses) were combined into a single tube with a final concentration of 7.5 ng/ul. The samples were sequenced using the 454-sequencing platform (using Titanium chemistry) at Engencore, University of South Carolina.

Sequences were analyzed with the software package “Quantitative Insights into Microbial Ecology” (QIIME). Sequences were removed if lengths were <200 or >1000 nt; contained ambiguous bases, primer mismatches, homopolymer runs in excess of six bases, or uncorrectable barcodes.

### 16S rRNA gene sequence analysis

The quality-checked sequences were denoised [47] and processed using QIIME [48]. Similar sequences were binned into operational taxonomic units (OTUs) using UCLUST [49], with a minimum pairwise identity of 97% and taxonomy was assigned to representative sequences using the Greengenes database [50].

### Fatty acid analysis

Serum, ileal and liver samples were collected from the Control (n = 4) and BCFA (n = 3) groups for FA analysis. Dietary lipids and ileal, liver and serum samples were extracted using a modified Bligh and Dyer method [51]. Ileal lipids were loaded on thin layer chromatography (TLC) plates (Silica gel G, 20×20, Analtech, Inc., Newark, DE). Plates were developed halfway in hexane∶diethyl-ether∶acetic acid (40∶60∶1, v∶v∶v), dried, and redeveloped with heptane∶diethyl-ether∶formic acid (80∶20∶1.8, v∶v∶v). Lipid bands were visualized in iodine vapor and the PL fraction was collected. Fatty acid methyl esters (FAME) for dietary lipids, ileal PL, liver and serum were prepared using 14% BF3/MeOH (Sigma Chemical, St. Louis, MO).

FAME analyses were performed as described previously [15], except that a BPX-70 column (25 m×0.22 mm×0.25 µm) (SGE, Austin, Tx) was used for gas chromatography (GC) analysis. FA levels are expressed as %, weight-for-weight (%w/w).

### Immunohistochemistry (IHC)

A 2-cm section of distal ileum was collected from each rat pup, and processed as described previously [42]. After deparaffinization and rehydration, sections were blocked in 5% bovine serum albumin (BSA) to prevent non-specific staining and incubated with goat anti-IL-10 polyclonal antibody (sc-1783; Santa Cruz Biotechnology, Santa Cruz, CA), followed by incubation with Alexa Fluor 594 conjugated anti-goat secondary antibody (Molecular Probes, Eugene, OR) for 1 h and coverslipped with Prolong gold anti-fade reagent with 4,6-diamidino-2-phenylindole (DAPI). An Olympus IX-70 inverted fluorescent microscope equipped with a 40× oil immersion objective was used to evaluate IL-10 staining. For quantification of fluorescence signal, digital images were saved in Adobe Photoshop CS5 and analyzed using ImageJ software (NIH, USA). Statistical comparison of densities in three samples from each experimental group was performed using one-way analysis of variance (ANOVA).

### Statistical analysis

A power calculation was based on mean NEC scores and standard deviations from previous work in which the mean and standard deviation scores for the control and tested agent were 1.61±0.70, and 1.16±0.50 respectively [42]. At least 21 animals were needed in each artificial feeding group for a significance level of 0.05 and 80% power using PROC POWER in SAS 9.2 (Cary, NC). We allocated 23 and 35 pups in the BCFA and control group, respectively. The Fisher Exact Test (*R* software [52]) was used to test the null hypothesis that the incidence of NEC is the same in the BCFA and control groups at p<0.05.

To test for differences in pup weights at the beginning and at the end of the study, and to test for differences in BCFA levels in ileum PL, serum and liver, ANOVA followed by Tukey's least significant difference (JMP 8, SAS Institute, Cary, NC) was used. ANOVA followed by Fisher's protected least significant difference (StatView for Macintosh, Abacus Concepts, Inc., Berkeley, CA) was used to analyze differences in gene expression and in IL-10 peptide IHC.

Differences in relative abundance of OTUs among the experimental groups were analyzed as described below using a mediation model [53] in two stages (*R* software [52]). In the first stage the response is the histology *score*, measured as a continuous variable which takes values between 0 and 4. The independent variables are the groups (BCFA, DF, or Control, with 11, 8, and 9 pups, respectively), and the bacteria count (BC). The following linear model was fitted:
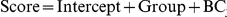
for each of the 29 OTUs (indexed by j). For each model, we obtained the p-value based on the F-statistic (testing whether none of the explanatory variables is significant, versus the alternative that at least one of the two explanatory variables is significant). The Bonferroni method was used to adjust the p-values for multiple testing of 29 OTUs. Controlling the Type-I error at a 5% level, a total of four models were significant, corresponding to *Pseudomonas aeruginosa*, *Clostridium*, *Bacillus subtilis*, and *Lactobacillus*.

In the second stage we assessed whether for these four bacteria, the BC variable was also affected by the treatment. The Generalized Linear Models (GLM) framework [54] was used to fit a Poisson model with a log link function:
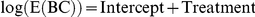
That is, the expected logarithm of bacteria count is a linear function of the treatment group.

As often observed when fitting a Poisson model to count data, we observed over-dispersion (the observed variance was larger than the mean). We adjusted the GLM fit accordingly.

Finally, to perform the three pairwise comparisons (between groups) and account for multiple testing, Tukey's honest significance test was used. After Tukey analysis, *Bacillus subtilis* and *Pseudomonas aeruginosa* were significantly different among the groups. The same statistical procedure was followed for the nine most abundant OTUs. The relative abundance of the various OTUs was positively skewed distribution and these data were natural log transformed for comparison. A significance level of p<0.05 was used.

## Results

### Clinical evaluation

Survival rate was similar among all groups with 87% (13/15), 89% (31/35), and 91% (21/23) in the DF, Control and BCFA groups, respectively. Body weights at the end of study were 8.06±0.24, 5.97±0.05, and 5.69±0.10 g (means ± SEM) for the DF, Control and BCFA groups, respectively. There were no significant differences in body weight between the Control and BCFA groups. The high relative weight of DF pups compared to the artificial feeding groups (*p*<0.05) was similar to previous studies [44], [52].

### NEC incidence

**Figure 3:**
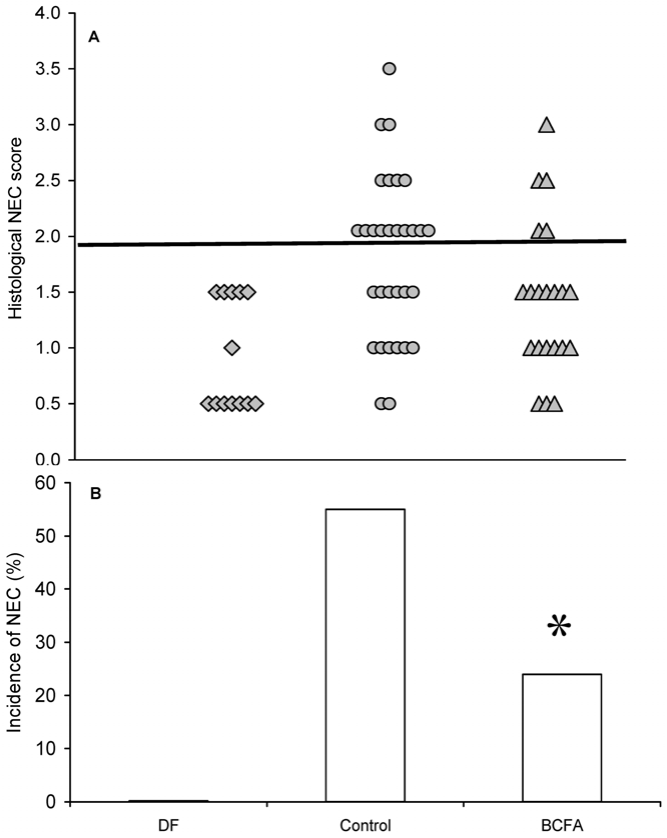
Histological scores and NEC incidence. (**A**) Histological scores and (B) incidence of NEC in rat pups in all experimental groups: DF, n = 13; Control, n = 31. BCFA n = 21 Pups were considered to have NEC if their histological score was ≥2; The incidence of NEC was significantly lower in the BCFA group compared to the Control group* *p*<0.05 Control vs. BCFA.

Histological NEC scores (Figure 3A) and the incidence of NEC (Figure 3B) in the experimental groups are plotted individually and summarized according to whether they had a score of 2 or greater indicative of NEC. Pups in the DF group do not normally develop NEC and no pup in the present study exhibited an abnormal intestinal architecture. In the Control group, 17 of 31 pups (55%) had a score of 2 or greater, indicating NEC injury. In contrast, the BCFA group had histological scores of 2 or greater in 5 of 21 pups (24%). The null hypothesis of no difference in NEC incidence between artificial feeding group was rejected (p = 0.044); BCFA reduced the incidence of NEC by 56% (55% vs 24%).

### Bacterial diversity

**Figure 4:**
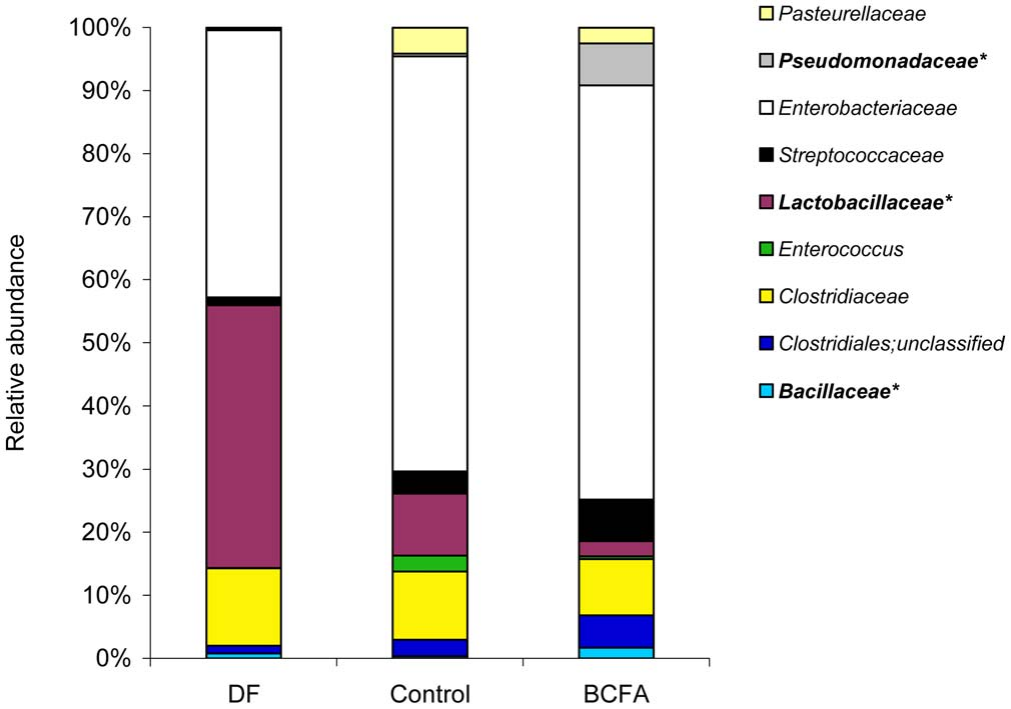
Distribution of the most abundant OTUs at the family level. The relative abundance (%) of the OTUs at the family level in the various experimental groups. DF (n = 8); Control (n = 9); BCFA (n = 11). *p<0.05 for group differences.

Nine OTUs at the family level predominated in the bacterial microbiota of all pups. *Bacillaceae* and *Pseudomonadaceae* families were significantly more abundant in the BCFA-fed pups compared to the Control group (Figure 4).

**Figure 5:**
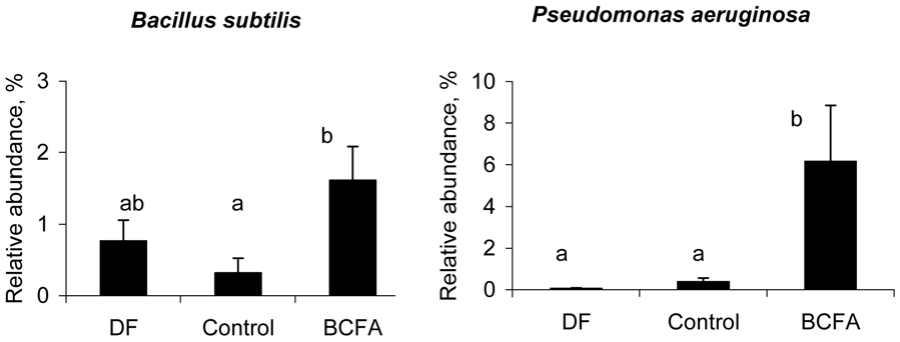
The relative abundance of *Bacillus subtilis* and *Pseudomonas aeruginosa*. The relative abundance (%; mean ± SEM) of *Bacillus subtilis* and *Pseudomonas aeruginosa* differed significantly among the experimental groups. DF (n = 8); Control (n = 9); BCFA (n = 11). Means not marked by the same letter are significantly different (p<0.05).

**Figure 6:**
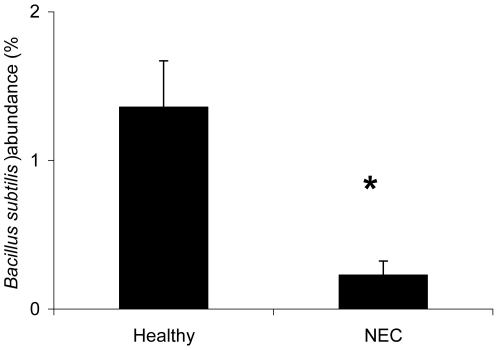
The relative abundance of *Bacillus subtilis* and health status. The relative abundance (%, mean ± SEM) of *Bacillus subtilis* in healthy animals and in animals with NEC. The relative abundance *of Bacillus subtilis* was higher in healthy animals regardless of diet group compared to animals with NEC. *p<0.05.

At the species level, the relative abundance of *Bacillus subtilis* was significantly higher in the BCFA group compared to the Control group, but not significantly different than the DF group (Figure 5). The relative abundance of *Pseudomonas aeruginosa* was significantly higher (p<0.05) in the BCFA-fed group compared to the Control and DF group. Moreover, the relative abundance of *B. subtilis* was significantly higher (p<0.05) in the healthy animals compared to sick animals (Figure 6). The difference was significant even when the DF group, all of which were healthy, was excluded from the analysis (not shown).

### BCFA in rat ileum PL, serum and liver

**Figure 7:**
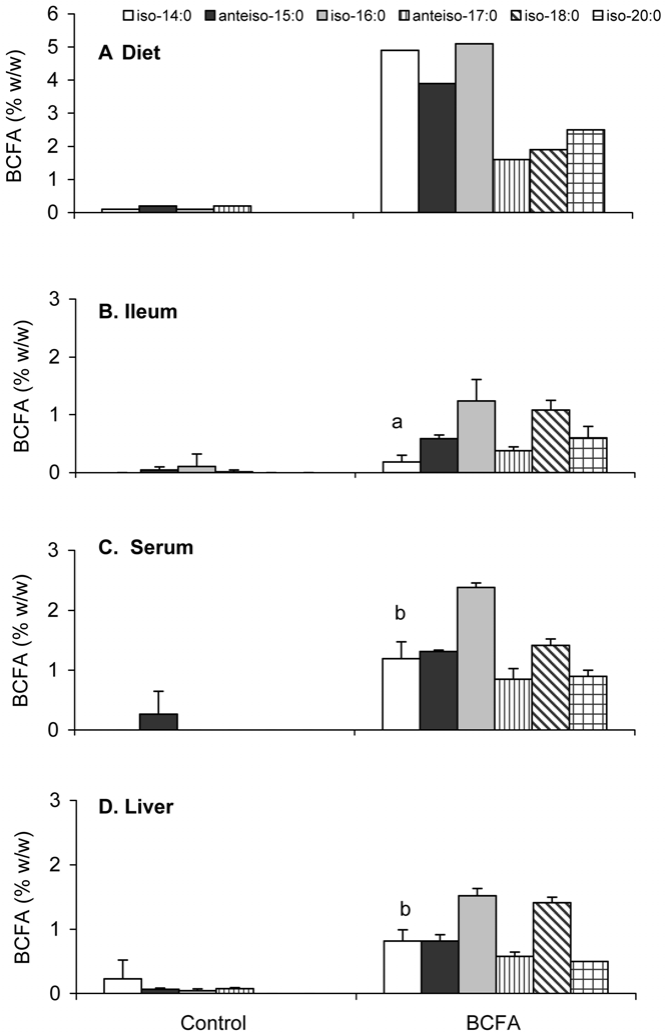
BCFA concentrations in the Control and BCFA groups. BCFA concentrations (%w/w) in (A) diets, and in a subset of Control and BCFA-fed pup (B) ileum PL, (C) serum, and (D) liver. Data are expressed as means ± SD (n≤4). Compared to their proportions in the diet, BCFA were incorporated in a selective manner in the ileum PL, serum and liver of the BCFA-fed pups. *Iso*-14:0, *iso*-18:0, and *iso*-20:0 are significantly altered as a fraction of BCFA in ileum, serum, and liver (p<0.05).

BCFA concentrations in the diets and in ileum PL fraction, serum and liver (%w/w; mean ± SD) of 3 pups without NEC from the BCFA group and 4 pups from the Control group are presented in **Figure 7**. Compared to their proportions in the diet (**Figure 7A**), BCFA were taken up by the ileum of the BCFA-fed pups (**Figure 7B**) and were incorporated in the ileum PL in a structure-selective manner: *iso-*14:0 and *anteiso-*15:0 were selected against and *iso-*18:0 was enriched in PL. This incorporation pattern was similar also in the serum and in the liver of the BCFA-fed animals (**Figures 7C and 7D**). Moreover, *iso*-14:0 was selected against in ileum compared to serum or liver. By contrast, BCFA concentrations in the ileum PL, serum and liver of pups from the Control group were at trace levels and may be due to endogenous synthesis from catabolized branched chain amino acids. Concentrations of non-branched FA in the ileum PL fraction as well as in the serum and in the liver were similar between the artificial feeding groups (data not shown), including oleic acid and linoleic acid, which differed in the diets. These data point to effects specific to BCFA and not to other FA in the diets.

### Interleukin (IL-10) mRNA and peptide localization

Ileal IL-10, mucins, and trefoil factor 3 mRNA levels probed by RT-PCR in ileum are presented in **Table 2**. IL-10 levels in the BCFA group were more than three-fold the values in the DF and Control groups. No other differences in mRNA were found between the artificially fed groups.

**Table 2:** Cytokine, Muc, and TFF3 mRNA levels in ileum of DF, Control and BCFA-fed neonatal rats^1^^,^ 2.

	DF	Control	BCFA
IL-10	1.0±0.1	1.0±0.1	3.2±1.5*
TGF-*β*	1.0±0.1	0.7±0.1†	0.7±0.1†
IL-18	1.0±0.1	1.3±0.1	1.7±0.1†
TNF-*α*	1.0±0.1	2.4±0.8	2.7±0.7†
IFN-*γ*	1.0±0.3	0.4±0.1†	0.7±0.4
Muc 2	1.0±0.1	0.5±0.1†	0.4±0.1†
Muc 3	1.0±0.1	1.6±0.3	1.2±0.2
Muc 4	1.0±0.1	0.3±0.8†	0.1±0.1†
TFF3	1.0±0.1	1.3±0.2	1.6±0.2

1 Mean steady-state mRNA levels of the Control-fed and BCFA-fed groups are expressed as fold changes relative to corresponding values for the DF group.

2 Data are means ± SEM; n = 10–23 pups per treatment.

*p<0.05 vs. Control and vs. DF.

† p<0.05 vs. DF.

**Figure 8:**
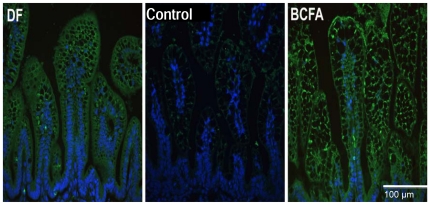
IHC images of IL-10. IHC images of IL-10 in healthy (score≤1.5) ileal sections from DF, Control, and BCFA pups. IL-10 is green, nuclear material is blue. Magnification: 400×. Compared to the Control group, IL-10 is increased in the BCFA group.

IHC was used to localize IL-10 peptide in pup ileum to further investigate gene upregulation in the BCFA group compared with the Control group, because gene expression changes are not strongly correlated with protein abundances. Typical IHC slides of ileal IL-10 peptide show that IL-10 is enhanced in the BCFA group (**Figure 8**). DF and BCFA pups displayed moderate IL-10 peptide in the villi cell cytoplasm. IL-10 signal intensity was significantly higher (fourfold, p<0.001) in BCFA and DF pup sections compared with the Control ileum.

## Discussion

BCFA are constituents of the GI tract of normal, healthy, term newborns [15]. In the present study, oral administration of BCFA was used to simulate the ingestion of BCFA-rich vernix by the human fetus. BCFA reduced the incidence of NEC among artificially-fed rat pups. There were no cases of NEC in pups from the DF group, presumably because this group enjoyed other nutritional benefits of dam-milk and the nurturing benefits of maternal care that may have enhanced the DF pups' resistance.

BCFA chain length distribution in vernix is from about C11 to C26, while that of meconium is from C16 to C26. Short BCFA entering the GI tract as AF-suspended vernix do not appear in meconium, indicating that BCFA are metabolized by the fetal gut. Here, dietary BCFA are incorporated in a structure-selective manner into the PL fraction of the ileum. Selection against short BCFA (<16 carbons) for incorporation into the PL fraction is consistent with our previous observation showing a systematic shift of BCFA distribution between vernix and meconium [15]. These results demonstrate that BCFA are not inert components of the GI tract, but are selectively incorporated into membrane lipids mediated by enterocytes, and are further transferred into circulation and to the liver.

The reduction in NEC incidence reported here is similar to most prophylactic treatments targeting NEC in experimental studies [53], [54], [55]. Our results are consistent with the hypothesis that altered FA membrane composition may change enterocyte resistance to initiating events associated with NEC. Moreover, the entry and possible accumulation or turnover of BCFA could contribute to signaling the GI tract that normal parturition is approaching and that the dramatic shift between AF and full reliance on breast-milk is imminent. An analogy can be drawn to the rise in blood cortisol that signals the lungs to increase surfactant production. This speculation is intriguing in part because lung surfactant PL appear to have a role in vernix particle entry into the AF [12], and thus levels of the two may be correlated for some infants.

The cecal microbiota results show that BCFA in formula, with total fat held constant, alters the relative abundance of bacterial families in the cecal microbiota. In particular, the relative abundance of each of two prominent BCFA-containing bacteria, *B. subtilis* and *P. aeruginosa*, was higher in the BCFA group compared to the Control group. BCFA levels in *B. subtilis* are 95% of the total FA [4], while those in *P. aeruginosa* can range from 0.3–16% of the total FA depending on growth conditions [56], [57]. Since the abundance of other types of bacteria was similar between treatments, it is likely that other factors beyond a supply of BCFA played a role in specifically enhancing some organisms over others and that pup-to-pup variability may also have masked more subtle changes in BCFA-reliant organisms.

*B. subtilis* is considered a commensal bacterium and is used as a probiotic [58]. Elevated levels of *B. subtilis* have been associated with improved macroscopic lesion scores, reduced levels of pro-inflammatory cytokines, and increased anti-inflammatory cytokine levels in colitis-induced animals compared to untreated colitis-induced animals [59]. *B. subtilis* levels have also been associated with improved performance (weight gain, intake, weaning age) and immune function (higher serum IgG) of preweaning calves, but did not affect IL-10 levels [60]. Though the relative abundance of *B.subtilis* was significantly higher in the BCFA group compared to the Control group, no correlation was found between the relative abundance of *B. subtilis* and cytokine mRNA levels, including IL-10 mRNA levels (data not shown). Nonetheless, the relative abundance of *B. subtilis* was higher in healthy animals regardless of treatment, suggesting it may play an elementary role in reducing the incidence of NEC. Further studies are necessary to establish whether *B. subtilis* may be a component of an effective prophylactic probiotic against NEC.

*P. aeruginosa* was significantly more abundant in the BCFA group compared to the Control group despite a lower NEC incidence in the BCFA group. In addition, *P. aeruginosa* levels were similar in the DF group and the Control group, despite the lack of NEC in the DF group. Reports of *P. aeruginosa* association with NEC are mixed [61], [62]. Recent data show that BCFA, specifically *iso*-14:0, *anteiso*-15:0 *iso*-16:0, and *anteiso*-17:0, repress motility in *P. aeruginosa* without significantly inhibiting its growth in liquid cultures [63]. A plausible explanation for the higher abundance of *P. aeruginosa* in the BCFA group, despite lower NEC incidence, is that BCFA in our study supported *P. aeruginosa* growth while repressing its motility and its consequent pathogenicity. Finally, *P. aeruginosa* may not be associated with NEC, regardless of treatment, under the conditions of our study.

Changes in IL-10 mRNA and protein also suggest possible mechanisms of BCFA protection against NEC. Ileal IL-10 expression was greater in the BCFA group relative to the Control group, as were IL-10 peptide levels in the IHC samples. IL-10 knockout mice spontaneously develop chronic enterocolitis throughout the intestinal tract [64]; early IL-10 administration prevented the development of enterocolitis in these mice, suggesting that IL-10 counterbalances effectors present in intestinal lesions [65]. Similarly, the protective effect of epidermal growth factor (EGF) against NEC was associated with a tripling of ileal mRNA IL-10 [66]; reduced NEC incidence in rat-milk-fed versus formula-fed pups was associated with a twofold increase of ileal IL-10 mRNA [52]. Others showed that subcutaneous administration of recombinant human IL-10 to the intestine of NEC-induced neonate rats reduced the severity of microscopic ileal lesions compared with untreated rats [67]. Thus, elevated ileal IL-10 mRNA here may mediate NEC reduction in the BCFA group, possibly by opposing proinflammatory effects. The higher mRNA levels of IL-10 in the BCFA group compared to the DF group can be explained by the lower levels of proinflammatory cytokines in the DF group, where no additional production of IL-10 was required to counteract them [52].

Proinflammatory cytokines are important factors in NEC pathogenesis [66], [68], [69]. Caplan et al [70] observed high plasma levels of TNF-α in infants with NEC, but no correlation between TNF-α levels and severity of disease. Here, ileal TNF-α gene expression was greater in the artificial feeding groups compared with DF pups. BCFA treatment did not specifically affect TNF-α mRNA levels. IFN-γ mRNA was significantly lower in the Control group compared to DF but the BCFA group was not different. The relationship of IFN-γ in NEC is ambiguous. Elevated intestinal IFN-γ levels have been reported in NEC patients [71], however no significant differences in IFN-γ were found between formula-fed-hypoxic NEC rats and non-hypoxic formula-fed groups with mild intestinal damage in a study similar to ours [72] and in separate studies ileal IFN-γ mRNA was not related to NEC development in neonatal rat pups [68].

Dietary *iso*- and *anteiso*-BCFA have not previously been studied in the context of human diet. This is the first study to investigate, and show, a protective effect of dietary BCFA on NEC. In humans, BCFA are ingested and absorbed in substantial amounts *in utero* as AF suspended vernix particles. Since vernix is uniquely human, interspecies comparison must be viewed with caution. Nevertheless, preclinical *in vivo* models are crucial for establishing safety and efficacy, and neonatal rats are an accepted model for NEC in human infants. In addition, we used six pure BCFA with chain length distribution from 14 to 20 carbons and in *iso*- and *anteiso*- branching types; vernix contains BCFA with a larger chain length range, 11 to 26 carbons, and with additional branching types [15]. Future research will delve more deeply into the influence of a larger variety of BCFA on intestinal microbial communities in NEC while seeking to replicate the beneficial effect of BCFA on NEC.

In conclusion, BCFA reduced the incidence of NEC by 56% in a neonatal rat pup model. The bacterial analysis showed that formula containing 20% of fat as BCFA, similar to the concentration in vernix, alters microbiota composition, in turn suggesting that BCFA are used by cecal bacteria. The greater abundance of *B. subtilis* and *P. aeruginosa* suggests that these or other BCFA-using organisms may participate in the BCFA moderating effect on NEC incidence. In addition, an increase in ileal IL-10 in the BCFA-fed group and a selective incorporation of these FA into membrane lipids may be also associated with a protective effect of BCFA against NEC.
